# Examining the factors influencing academic performance of medical technology students in e-learning: A questionnaire survey

**DOI:** 10.1371/journal.pone.0311528

**Published:** 2024-12-12

**Authors:** Ding-Ping Chen, Ai-Ling Hour, Kuo-Chien Tsao, Chung-Guei Huang, Wei-Tzu Lin, Fang-Ping Hsu

**Affiliations:** 1 Department of Laboratory Medicine, Chang Gung Memorial Hospital at Linkou, Taoyuan, Taiwan; 2 Medical Biotechnology and Laboratory Science, Chang Gung University, Taoyuan, Taiwan; 3 Department of Life Science, Fu Jen Catholic University, New Taipei City, Taiwan; Endeavour College of Natural Health, AUSTRALIA

## Abstract

In an era of rapid digital development, e-learning has become a significant trend in the educational field. Medical technology students need to acquire extensive theoretical knowledge and practical skills. E-learning can enhance learning experiences, improving students’ understanding and application abilities. This study examined the impact of learning motivation, learning approaches, and learning burnout on the academic performance of medical technology students in an e-learning environment. This study conducted a quantitative survey on 37 medical technology students. The questionnaires included learning motivations, learning methods, and burnout, and responses provided on a 5-point Likert scale. First, the 37 students were categorized into three groups based on their academic performance. Then, differences between these groups were analyzed using the Kruskal-Wallis test, and correlations between academic performance and questionnaire variables were calculated using Spearman correlation analysis. It revealed that motivation varies among different academic performance levels. Furthermore, in the high-grade group, self-efficacy (r = -0.502, p = 0.047), monitoring studying (r = 0.494, p = 0.032), and emotional exhaustion (r = 0.514, p = 0.024) were correlated to academic performance. In the middle-grade group, self-efficacy for learning and performance was correlated to academic performance (r = 0.858, p = 0.001). In the low-grade group, academic performance was correlated to depth approach (r = 0.826, p = 0.022) and organized studying (r = 0.833, p = 0.020). This study, through a quantitative survey, found significant differences in learning motivation, learning methods, and learning burnout among medical technology students with different academic performance levels. High-grade students demonstrated higher levels of emotional exhaustion, which may reflect feelings of overextension and academic burnout in e-learning environments; the academic performance of the middle-grade group was related to intrinsic motivation; and low-grade students showed a stronger correlation between their learning methods in e-learning and their academic performance. These insights underscore the necessity for personalized learning strategies to enhance educational outcomes effectively.

## Introduction

In today’s rapidly advancing digital era, e-learning has become a significant trend in the educational field. With technological progress and the widespread availability of the internet, traditional teaching methods are gradually being replaced by e-learning [1]. Since many of the learning components for medical technology interns involve hands-on instructional courses in medical education, e-learning with the ability to watch videos is expected to provide good learning outcomes within them, compared to text only [2]. Medical technology students need to acquire extensive theoretical knowledge and practical skills, which traditional teaching methods may sometimes struggle to meet. For instance, laboratory protocols, complex concepts and techniques in anatomy or physiology, and case studies involving data interpretation can all be enhanced through e-learning [3, 4]. E-learning can enrich students’ learning experiences, thereby improving their understanding and application abilities. Enhancing learning experiences, particularly for medical technology students, positively impacts learning outcomes [5]. Moreover, in the previous study, we found that medical technology students’ learning effectiveness primarily depended on three factors: “extracurricular studies”, “willingness to cooperate”, and “weakened motivation due to uncertainty”. Among that, a positive correlation between “extracurricular studies” and “willingness to cooperate” with academic performance, while a negative correlation was observed between academic performance and “weakened motivation due to uncertainty”. This was published in the BMC Medical Education journal in 2023 [6]. The electronic learning (e-learning) mode offers the advantages of freedom, diversity, and flexibility from geographical and time constraints [7]. For example, platforms like MOODLE have been widely adopted in educational institutions to provide a structured yet flexible learning environment, and they support various interactive features such as forums, quizzes, and assignments, which can enhance student engagement and promote collaborative learning [8]. Moreover, there are numerous methods available to enhance interactivity in online learning, such as gamification [9]. Therefore, we believe e-learning can promote extracurricular learning and collaborative discussions to enhance medical technology student’s academic performance. Especially after the global pandemic outbreak, distance teaching and online learning have flourished. The e-learning mode can be used to train medical technology interns, providing cost-effective education. This method of teaching is not restricted by location, especially in situations involving a large number of students who may be spread out across different areas. This makes it an indispensable tool during times of pandemic for course instruction, unlike traditional teaching methods [10, 11].

Students with strong learning motivation typically perform better academically because they have a positive attitude and intrinsic drive toward learning [12, 13]. Since there are various theories of motivation, including intrinsic goal orientation, extrinsic goal orientation, and affective factors [14], we used the Motivated Strategies for Learning Questionnaire (MSLQ) to assess students’ motivation. Effective learning methods help students better understand and master course content, thereby improving learning efficiency [15, 16]. We used the Approaches to Learning and Studying Inventory (ALSI) to assess students’ learning methods. Learning burnout can negatively impact academic performance, leading to decreased interest in learning and poor grades [17]. We used the Maslach Burnout Inventory–Student Survey (MBI-SS) to assess students’ burnout. These three factors—learning motivation, learning methods, and learning burnout—are commonly used to evaluate their correlation with academic performance. Thus, they were chosen to assess the factors influencing the learning outcomes of medical technology students in e-learning environments. In our previous research, we also used these three questionnaires to comprehensively investigate potential factors affecting the academic performance of medical technology students. Therefore, in this study, we simultaneously examine learning motivation, learning methods, and learning burnout to investigate the factors influencing the academic performance of medical technology students in e-learning.

However, the specific impact of e-learning on the academic performance of medical technology students in Taiwan requires further research. Previous studies have shown that e-learning can enhance students’ interest in learning and their self-directed learning abilities [18], but its effectiveness may vary across different subjects and educational environments. Moreover, while some studies have investigated the correlation between academic performance and factors such as motivation, learning methods, and burnout, none have specifically analyzed these factors across different performance levels in the context of medical technology education in Taiwan. This study addresses this gap by investigating the factors affecting academic performance in e-learning platforms across three grade levels, providing empirical support for improving future educational models and optimizing teaching resources in this specific context.

## Materials and methods

### Objectives

The purpose of this study was to investigate the learning motivation, learning methods, and burnout of students with different grade levels in e-learning to understand what factors affect their academic performance.

### Design

These participants were first divided into three groups according to their academic performance levels. Then, the learning motivation, learning approaches, and burnout on e-learning were investigated to explore the influencing factors on students’ academic performance. The e-learning program under consideration is a fully online curriculum, originally designed as such and not a temporary response to the COVID-19 pandemic. We used MOODLE [19], one of the most widely adopted free and open-source e-learning platforms, to conduct our e-learning instruction. The online teaching format includes both asynchronous and synchronous components, offering flexibility for students. Importantly, the program is not exclusively conducted online; it incorporates both in-person and non-face-to-face elements. Contrary to a traditional didactic lecture approach, the e-learning program features a diverse instructional methodology. While didactic lectures are included, the curriculum goes beyond, incorporating interactive and active learning strategies to engage students. This multi-modal approach aims to enhance the overall learning experience and adapt to the varied preferences and needs of the student body. The unexpected shift to online learning during the pandemic underscored the program’s resilience and efficacy, demonstrating its adaptability to unforeseen circumstances and providing valuable support to students during challenging times.

### Participants

This study included 37 senior medical technology students interning at the Department of Laboratory Medicine of Chang Gung Memorial Hospital, consisting of 27 females and 10 males. These participants were included from 2024/3/8 to 2024/5/30. They were informed to do this test, and they could decide whether to participate or not. Their academic performances were evaluated based on their grades in hematology and molecular testing (including experiments and internships). The internship scores are subjectively evaluated by teachers based on students’ performance and regular assessments. Due to the subjective nature of these evaluations, there is bias, variability, and potential for grade inflation or deflation in the internship scores. Teachers may unintentionally inflate or deflate grades based on their expectations or perceptions, leading to an inaccurate representation of students’ actual performance and learning outcomes. To assess students’ performance more accurately, we quantified these evaluation results and categorized students into three levels: high, middle, and low scores. Therefore, these students were categorized into three groups based on their middle academic performance: the high-grade group (90–99, n = 19), the middle-grade group (81–89, n = 11), and the low-grade group (under 80, n = 7), to further examine factors correlated to student’s academic performance.

### Questionnaires

The questionnaires were distributed in paper format, written in Chinese, and there was no time limit for completing them. The questionnaires analyzed intrinsic factors affecting the learning effectiveness of e-learning platforms using the Motivated Strategies for Learning Questionnaire (MSLQ) and the Maslach Burnout Inventory-Student Survey (MBI-SS) and external factors affecting the learning effectiveness of e-learning platforms using the Approaches to Learning and Studying Inventory (ALSI). Responses are provided on a 5-point Likert scale, with higher values indicating a stronger inclination towards the trends cited. Additionally, the scores of each subcategory were summarized as comprehensive scores for analysis.

### Motivated Strategies for Learning Questionnaire (MSLQ)

This questionnaire was adapted from the motivation section of the "Revised Motivated Strategies for Learning Questionnaire," which was published by Wu and Cherng in 1992 [20]. It was used to measure the motivation of medical technology students on the e-learning platform. The questionnaire consists of 35 items with a total of 8 subcategories.

1. **Value components**: learners’ beliefs regarding the importance and value of learning tasks and their reasons for engaging in learning tasks, including three subcategories: intrinsic goal orientation (4 items), extrinsic goal orientation (4 items), and task value (6 items).
2. **Expectancy components**: beliefs about one’s ability or skill to complete a learning task and beliefs about control and expectations of success in learning tasks, including three subcategories: control beliefs about learning (8 items), self-efficacy for learning and performance (5 items), and expectancy (3 items).
3. **Affective components**: emotional responses to learning tasks, learning outcomes, or one’s learning abilities, primarily measured by test anxiety. It refers to the emotions and feelings a learner experiences before or during an exam due to worry or fear, including two subcategories: cognitive interference (3 items) and emotionalism (2 items).

### Approaches to Learning and Studying Inventory (ALSI)

This questionnaire was adapted from the " Approaches to Learning and Studying in Medical Students" by Mattick in 2004 [21]. It was used to measure the learning and study methods perceived by medical technology students after using the e-learning platform. It consists of 18 items with a total of 5 subcategories.

1. **Deep approach** (6 items): It measures the degree to which students understand the content of the learning process and the use of teaching materials. Those with high scores have qualities such as achieving mastery through a comprehensive study of the subject.
2. **Surface approach** (4 items): Similar to the deep approach, it is used to measure the degree to which students understand the content of the learning process and the use of teaching materials. However, those with high scores in this subscale have characteristics such as the inability to effectively integrate knowledge content. They tend to learn out of fear of failure, and their approach is rote.
3. **Monitoring studying** (4 items): It measures students’ understanding of knowledge during the learning process. Those with high scores have characteristics such as monitoring their understanding of knowledge.
4. **Organized studying** (2 items): It measures students’ ability to integrate and manage their learning process. Those with high scores have characteristics such as integration and time management.
5. **Effort management** (2 items): It measures students’ effort in the learning process. Those with high scores have characteristics such as organized learning.

### Maslach Burnout Inventory Student Survey (MBI-SS)

This questionnaire was adapted from Schaufeli’s " Maslach Burnout Inventory Stu-dent Survey" [19] and is used to measure students’ learning burnout regarding the use of e-learning platforms. It consists of 15 items with a total of 3 subcategories.

1. **Emotional exhaustion** (5 items): exhaustion of emotional resources due to demanding interpersonal interactions with others.
2. **Cynicism** (4 items): An attitude of passivity, cynicism, and disillusionment towards learning.
3. **Academic efficacy** (6 items): Tendency to evaluate one’s achievements negatively.

The content of the above questionnaires was translated and developed based on the literature [20–22]. The reliability and validity of these questionnaires in Chinese form have been tested and are widely used [23, 24]. The complete survey questions are shown in S1 Table.

### Ethical approval

Students were informed that they could decide for themselves whether to take this test. No patient identifying or personal information was collected as part of this study. Research data were delinked and stored in the Chang Gung Memorial Hospital laboratory. All participants had signed informed consent forms before completing the questionnaires. This study was reviewed and approved by the Institutional Review Board of Chang Gung Hospital; its approval ID was 202300714B0. All methods were performed in accordance with the relevant guidelines and regulations.

### Statistical analysis

Each question/item of the questionnaire was coded and analyzed as an ordinal explanatory variable. In addition, the scores within subcategories were summed to analyze. The Kruskal-Wallis test with Dunn’s post-test was conducted to assess differences in the scores of each question/ subcategory between the different performance groups. The Spearman correlation analysis was used to calculate the correlation coefficients between academic performance and the scores of each questionnaire/ subcategory.

## Results

The 37 students were divided into three groups based on their grades: high-grade group (91–99, n = 19), middle-grade group (81–89, n = 11), and low-grade group (under 80, n = 7).

Analyzing differences in question scores and comprehensive scores across different academic performance levels (Table 1), the findings showed that the scores of MSLQ_3 (p = 0.042), MSLQ_7 (p = 0.005), and MSLQ_14 (p = 0.043) were significantly different among three academic performance levels, in which the scores of MSLQ_3 in the high-grade group (1.79±0.63) had a significant difference with low-grade group (3.00±1.29) (p = 0.018); the scores of MSLQ_7 in the low-grade group (3.86±0.69) had significant differences with the high grades group (2.79±0.54) and the middle-grade group (2.73±0.79), p = 0.015 and p = 0.003, respectively; the scores of MSLQ_14 in the high-grade group (3.79±0.54) had a significant difference with the middle-grade group (3.18±0.60) (p = 0.023). The comprehensive scores of 16 subcategories had no difference among these three groups.

**Table 1:** Items showing significant score differences among different performance groups.

Item			p^a^	Grade levels		p^b^
Coded item	Description	Subcategory				
MSLQ_03	When reading or submitting assignments on the e-learning platform, I may think about how inferior I am compared to other students.	cognitive interference	0.042*	A	B	0.085
					C	0.018*
				B	C	0.672
MSLQ_07	I am confident that I can understand the most challenging parts of the e-learning platform course.	self-efficacy for learning and performance	0.005*	A	B	0.703
					C	0.015*
				B	C	0.003*
MSLQ_14	If possible, I want my grades on the e-learning platform course to be better than most of the students in the class.	extrinsic goal orientation	0.043*	A	B	0.023*
					C	0.395
				B	C	0.425

p^a^: p-value of Kruskal-Wallis test; p^b^: p-value of Dunn’s test; A: high-grade group; B: middle-grade group; C: low-grade group. *: significance (p<0.05).

Further investigation found specific correlations between question/subcategory scores and academic performance in each grade level group. In a correlation analysis between the scores of each question/subcategory and the academic performance in the high-grade group, it was found that the scores of MSLQ_22 and MBI_08 were negatively related to academic performance (r = -0.461, p = 0.047). In addition, the comprehensive scores of “self-efficacy for learning and performance” (r = -0.502, p = 0.028), “monitoring studying” (r = 0.494, p = 0.032), and “emotional exhaustion” (r = 0.514, p = 0.024) were also had correlations (Table 2).

**Table 2:** Items correlated with academic performance in the high-grade group.

Item			Spearman Test	
Coded item	Description	Subcategory	r	p-value
MSLQ_22	I am confident in performing well on assignments in the e-learning platform course.	self-efficacy for learning and performance	-0.461	0.047
MSLQ	self-efficacy for learning and performance		-0.502	0.028
ALSI	monitoring studying		0.494	0.032
MBI-SS	emotional exhaustion		0.514	0.024

MSLQ: Motivated Strategies for Learning Questionnaire; ALSI: Approaches to Learning and Studying Inventory; MBI-SS: Maslach Burnout Inventory Student Survey; “*”:p<0.05

In the middle-grade group, it was found that the scores of MSLQ_6 (r = 0.798, p = 0.003), MSLQ_9 (r = 0.694, p = 0.018), MSLQ_11 (r = 0.641, p = 0.034), MSLQ_20 (r = 0.630, p = 0.038), MSLQ_26 (r = 0.636, p = 0.035), and ALSI_10 (r = 0.648, p = 0.031) were related to academic performance. Furthermore, the comprehensive score of “self-efficacy for learning and performance” was related to academic performance (r = 0.858, p = 0.001) (Table 3).

**Table 3:** Items correlated with academic performance in the middle-grade group.

Item			Spearman Test	
Coded item	Description	Subcategory	r	p-value
MSLQ_06	I believe that I will achieve excellent grades in the e-learning platform.	expectancy	0.798	0.003
MSLQ_09	I worry about the course content that I haven’t learned yet while doing assignments on the e-learning platform.	cognitive interference	0.694	0.018
MSLQ_11	It is essential to master the content of the e-learning platform’s course.	task value	0.641	0.034
MSLQ_20	If I work hard enough, then I will understand the content of the e-learning platform.	control beliefs	0.630	0.038
MSLQ_26	In the e-learning platform, if I have the opportunity to choose the learning content, I would choose the content from which I can learn, even if it doesn’t guarantee good grades.	intrinsic goal orientation	0.636	0.035
ALSI_10	When I’m reviewing information on the e-learning platform, I can quickly synthesize my perspectives.	monitoring studying	0.648	0.031
MSLQ	self-efficacy for learning and performance		0.858	0.001

MSLQ: Motivated Strategies for Learning Questionnaire; ALSI: Approaches to Learning and Studying Inventory

In the low-grade group, the scores of the “depth approach” (r = 0.826, p = 0.022) and “organized studying” (r = 0.833, p = 0.020) were positively related to academic performance (Table 4).

**Table 4:** Items correlated with academic performance in the low-grade group.

Item			Spearman Test	
Coded item	Description	Subcategory	r	p-value
MSLQ_05	I believe I can apply the content I’ve learned from the e-learning platform’s courses to other courses.	task value	0.770	0.043
MSLQ_17	I am confident that I can understand the most complex content in the courses designed by the teacher on the e-learning platform.	self-efficacy for learning and performance	0.825	0.022
MSLQ_22	I am confident in performing well on assignments in the e-learning platform course.	self-efficacy for learning and performance	0.825	0.022
MSLQ_28	I enjoy the content of the e-learning platform’s courses.	task value	0.770	0.043
ALSI_03	I’m beginning to understand the significance of learning through the e-learning platform.	deep approach	0.822	0.023
ALSI_04	I put a lot of effort into my studies on the e-learning platform.	effort management	0.829	0.021
ALSI_05	What I’ve learned on the e-learning platform often lingers in my mind.	surface approach	0.829	0.021
ALSI_06	The knowledge I acquire on the e-learning platform can be connected to practical applications	deep approach	0.808	0.028
ALSI_07	My learning approach on the e-learning platform is systematic and organized.	organized studying	0.770	0.043
ALSI_10	When I’m reviewing information on the e-learning platform, I can quickly synthesize my perspectives.	monitoring studying	0.825	0.022
ALSI_11	I will plan my e-learning platform study time and make the most of it.	organized studying	0.825	0.022
ALSI_14	I will search for relevant information outside of the e-learning platform.	monitoring studying	0.762	0.046
ALSI	deep approach		0.826	0.022
ALSI	organized studying		0.833	0.020

MSLQ: Motivated Strategies for Learning Questionnaire; ALSI: Approaches to Learning and Studying Inventory

## Discussion

Here, in addition to investigating the factors that influence academic performance on e-learning platforms, we were also inversely examining the students’ evaluation and gains from e-learning among students with varying degrees of academic performance.

Based on the levels of academic performance, three questionnaire items from the MQLS showed significant differences among the high, middle, and low-grade groups: MSLQ_03 (cognitive interference), MSLQ_07 (self-efficacy for learning and performance), and MSLQ_14 (external goal orientation). The scores of MSLQ_03 showed a significant difference between the high and low grades group. The students in the low-grade group believed that their performance in reading or submitting assignments on the e-learning platform was poorer compared to other students. Research indicated that students’ self-confidence could impact their learning motivation [25]. Therefore, students with lower grades may believe that their performance on e-learning platforms may not be as good as others because of their relatively poor performance in traditional education. The scores of MSLQ_03 were significantly higher in the low grades group than in other groups, indicating that they believed they could comprehend the most challenging aspects of the e-learning platform’s course content and have a preference for the content on the e-learning platform. Previous research has indicated that students with “Learning Disabilities” were suitable for e-learning [26]. Although "Learning Disabilities" in that study refers to conditions that can affect one or more elementary and/or secondary school academic abilities, our study also suggested that students with lower academic performance were better suited for the current content of e-learning. The scores of MSLQ_14 showed a significant difference between the high and middle-grade groups. The students with better academic performance tended to have a higher external goal orientation, believing that their e-learning performance should surpass that of their peers. Currently, there were several studies indicating that motivation was indeed correlated with academic performance [27, 28]. In general, those with lower academic performance often exhibit lower self-regulation abilities, such as a lack of motivation and inefficient resource management [29]. Therefore, it should be possible to assist middle-grade students in enhancing their learning motivation to improve their learning effectiveness in e-learning.

In the comprehensive scores analysis, the findings showed that the scores of “self-efficacy for learning and performance” were significantly negatively correlated with academic performance in the high-grade group, while the scores of “monitoring studying” and “emotional exhaustion” had a positive correlation. This could be explained by the fact that individuals with better academic performance may lack confidence in their performance on the e-learning platform’s courses and experience increased stress and their understanding of the content on the e-learning platform was likely reflected in their grades. Moreover, it was suggested that high-grade students may experience higher levels of stress and anxiety, which paradoxically seems linked to their academic success. This finding challenges the conventional notion that emotional exhaustion is purely detrimental and suggests that high-performing students might be using their stress as a motivational tool or adaptive strategy. This may be because they have already achieved good learning outcomes through traditional teaching methods and are concerned that changing their learning approach and a less robust content delivery could hurt their grades. This explanation has been explained in a study conducted in Korea, where the transition to online courses has led to an overall decrease in learning performance [30]. Although this course was originally designed as an e-learning program and not in response to the COVID-19 pandemic, it happened to coincide with the ongoing global health crisis. During this period, students faced numerous additional variables that could potentially contribute to poorer academic performance. These factors include personal illness, caregiving responsibilities for unwell family members, homeschooling family members, the pervasive fear and anxiety caused by the pandemic, and a lack of adequate technological resources, leading to reduced engagement in e-learning and subsequent academic challenges. In contrast, students with middle grades showed strong correlations between self-efficacy and academic performance, indicating that confidence in one’s ability to succeed plays a crucial role in their success. A study has found that online learning is well-suited for self-motivated, proactive learners [31]. This was similar to our results, we indicated that the self-efficacy motivation of medical technology students was positively correlated with their academic performance. For low-grade students, the study revealed a significant relationship between organized studying and academic outcomes, emphasizing the importance of structured study methods in achieving better performance. However, they may have misunderstood the content or have poorer organizational and time management skills. Therefore, it was recommended that educators incorporate more interactive learning methods into e-learning course designs, such as team-based learning, as it could be more advantageous for students with lower grades in terms of knowledge acquisition than for students with better performance [32]. There was no significant correlation between the comprehensive scores within MBI-SS and academic performance levels. This may be because the e-learning platform is a relatively novel teaching model, and as a result, students generally do not experience burnout from it. Research has found that online learning is well-suited for individuals who can effectively organize their study plans [33]. We also observed this, particularly among lower-performing medical technology students, whose academic performance was more closely related to their learning methods.

In education and learning, e-learning involves educators delivering course materials through digital learning platforms over the Internet. Students engage in synchronous or asynchronous learning through online connections. Furthermore, by incorporating effective instructional design and facilitating real-time interactions with teachers and peers, e-learning aims to enhance the overall learning outcomes [34]. This study only explored the correlations between learning motivation/ learning approaches/ burnout and academic performance levels. Because e-learning systems are typically established by educators, they are currently in the developmental stage, and as a result, the content is often basic and standardized. Therefore, there are usually challenges in the initial stages, especially for students with lower adaptability. Furthermore, e-learning primarily provides knowledge based on theoretical foundations, and there may be limitations when it comes to applying what you’ve learned. Hence, there is still significant room for improvement in e-learning. It is hoped that in the future, a personalized e-learning system can be designed. Before learning, the system could use questionnaires to automatically provide tailored instructional content, thus enhancing learning effectiveness. It is crucial to emphasize that our recommendation is not merely about tailoring courses for individual learners. Instead, we advocate that, in the overall course design, motivation can be enhanced by considering the value components, expectancy components, and affective components of students across different academic levels, ensuring that all students can benefit. This approach is not only practical but also a fundamental part of best practices in educational design, encouraging educators to better adapt to the diversity among students. These findings suggested that developing personalized learning strategies tailored to students of different performance levels could effectively enhance learning outcomes.

The strengths of this study include our comprehensive assessment of students’ acceptance of the e-learning platform based on their learning motivation, learning approaches, and burnout. We also examined the acceptance of e-learning platform content among students with different academic performance levels. This study had limitations. The sample size was the major limitation of this study, especially as it became even smaller after dividing the students into three groups based on their performance, which led us to choose non-parametric methods as they are more appropriate for analyzing skewed data. Given the specific population of medical technology students and the constraints of our study period, 37 participants were a feasible and realistic number. We aimed to include all available students within the given constraints, rather than using a selective sampling method. However, since our grading criteria include subjective teacher-assigned internship scores in addition to objective exam results, we believed categorizing students into high, middle, and low-grade groups helped mitigate the potential for grade inflation or deflation. This approach aimed to balance the subjective elements of the evaluation and provide a more accurate assessment of student performance. Moreover, the validity and reliability of the Chinese version of the ALSI questionnaire have not been validated. Additionally, another challenge faced is the incompatibility of e-learning to teach certain skills, as some job-related skills such as staining, slide preparation, microscope usage, hematology, and histotechnology techniques are difficult to impart through online learning systems. These skills require equipment, hands-on practice, and experience, and can only be supplemented by simulated training for practical experimentation. To address the limitations identified in this study and to build on the findings, future research should focus on several key areas. Increasing the sample size is crucial to enhance statistical power and generalizability; a larger and more diverse participant pool could provide more comprehensive insights into the factors affecting academic performance. Additionally, employing objective and standardized evaluation metrics alongside subjective assessments could reduce potential grading biases and offer a clearer picture of student performance. Validation of the Chinese version of the Approaches to Learning and Studying Inventory (ALSI) and other measurement tools is also essential to ensure their reliability and applicability. Furthermore, exploring hybrid models that combine e-learning with in-person practical sessions could address the limitations of online education in teaching hands-on skills. Such studies could investigate effective ways to integrate practical training into e-learning platforms, thereby enhancing the overall educational experience and outcomes.

This was the first systematic exploration of the acceptance of e-learning platforms among students with different levels of academic performance. These findings underscore the importance of considering diverse factors influencing students’ motivation and performance across different academic levels, providing valuable insights for tailoring educational approaches to individual needs. It was hoped that in the future, personalized e-education environments can be developed to align with students’ preferences and enhance their motivation to promote learning effectiveness.

## Conclusion

Our study provided comprehensive insights into the acceptance of e-learning platforms among medical technology students with varying levels of academic performance. We identified significant differences in learning motivation, approaches, and burnout across different performance levels, highlighting the need for personalized learning strategies. The implications of these findings suggestted that personalized interventions focusing on enhancing self-efficacy, organizational skills, and stress management could be beneficial. Educational strategies should be tailored to address the specific needs of students at different performance levels to optimize learning outcomes in e-learning environments.

## Supporting information

S1 Table The complete survey questions. (DOCX)
