# Supplementary material for: Examining the factors influencing academic performance of medical technology students in e-learning: A questionnaire survey
Source: PLoS One. 2024 Dec 12;19(12):e0311528. doi: 10.1371/journal.pone.0311528 (PMC11637255; doi:10.1371/journal.pone.0311528)
Supplement: S1 Table — (DOCX) [file pone.0311528.s001.docx]

**Supplementary Table 1.** **The complete survey questions**

| **Questionaries** | **Coded item** | **Subcategories** | **Strongly disagree** | **Disagree** | **Neutral** | **Agree** | **Strongly agree** |
| --- | --- | --- | --- | --- | --- | --- | --- |
|  |  |  | **1** | **2** | **3** | **4** | **5** |
| **B.    Motivated Strategies for Learning Questionnaire, MSLQ** |  |  |  |  |  |  |  |
| In the e-learning platform, I prefer content that challenges me because it helps me learn new things. | MSLQ_01 | intrinsic goal orientation |  |  |  |  |  |
| If I use the e-learning platform effectively, I can learn the content of this course. | MSLQ_02 | control beliefs |  |  |  |  |  |
| When reading or submitting assignments on the e-learning platform, I may think about how inferior I am compared to other students. | MSLQ_03 | cognitive interference |  |  |  |  |  |
| If I don't understand the content of the course on the e-learning platform, it's because the course is too difficult. | MSLQ_04 | control beliefs |  |  |  |  |  |
| I believe I can apply the content I've learned from the e-learning platform's courses to other courses. | MSLQ_05 | task value |  |  |  |  |  |
| I believe that I will achieve excellent grades in the e-learning platform. | MSLQ_06 | expectancy |  |  |  |  |  |
| I am confident that I can understand the most challenging parts of the e-learning platform course. | MSLQ_07 | self-efficacy |  |  |  |  |  |
| Getting good grades in e-learning platform courses is what I'm most satisfied with. | MSLQ_08 | extrinsic goal orientation |  |  |  |  |  |
| I worry about the course content that I haven't learned yet while doing assignments on the e-learning platform. | MSLQ_09 | cognitive interference |  |  |  |  |  |
| If I haven't learned the content of the e-learning platform, it's my problem. | MSLQ_10 | control beliefs |  |  |  |  |  |
| It is essential to master the content of the e-learning platform's course. | MSLQ_11 | task value |  |  |  |  |  |
| The most important thing right now is to improve my overall semester grade in the e-learning platform course. | MSLQ_12 | extrinsic goal orientation |  |  |  |  |  |
| I am confident that I can learn the fundamental concepts taught in the e-learning platform course. | MSLQ_13 | self-efficacy |  |  |  |  |  |
| If possible, I want my grades on the e-learning platform course to be better than most of the students in the class. | MSLQ_14 | extrinsic goal orientation |  |  |  |  |  |
| If I understand the content of the e-learning platform course, it is mainly due to the teacher's influence. | MSLQ_15 | control beliefs |  |  |  |  |  |
| When reading or submitting assignments on the e-learning platform, I think about the consequences of poor grades. | MSLQ_16 | cognitive interference |  |  |  |  |  |
| I am confident that I can understand the most complex content in the courses designed by the teacher on the e-learning platform. | MSLQ_17 | self-efficacy |  |  |  |  |  |
| In e-learning platform courses, I prefer content that piques my curiosity, even if it's more challenging. | MSLQ_18 | intrinsic goal orientation |  |  |  |  |  |
| I'm very interested in the content of e-learning platform courses. | MSLQ_19 | task value |  |  |  |  |  |
| If I work hard enough, then I will understand the content on the e-learning platform. | MSLQ_20 | control beliefs |  |  |  |  |  |
| I feel uncomfortable and uneasy when reading or submitting assignments on the e-learning platform. | MSLQ_21 | emotionalism |  |  |  |  |  |
| I am confident in performing well on assignments in the e-learning platform course. | MSLQ_22 | self-efficacy |  |  |  |  |  |
| I anticipate performing well in the e-learning platform course. | MSLQ_23 | expectancy |  |  |  |  |  |
| The most satisfying thing for me in the e-learning platform course is trying to thoroughly understand the course content. | MSLQ_24 | intrinsic goal orientation |  |  |  |  |  |
| I believe that the e-learning platform's learning content is beneficial to me. | MSLQ_25 | task value |  |  |  |  |  |
| In the e-learning platform, if I have the opportunity to choose the learning content, I would choose the content from which I can learn, even if it doesn't guarantee good grades. | MSLQ_26 | intrinsic goal orientation |  |  |  |  |  |
| If I don't understand the content of the e-learning course well enough, it's because I haven't worked hard enough. | MSLQ_27 | control beliefs |  |  |  |  |  |
| I enjoy the content of the e-learning platform's courses. | MSLQ_28 | task value |  |  |  |  |  |
| Understanding the content of the e-learning course is very important to me. | MSLQ_29 | task value |  |  |  |  |  |
| If I didn't learn the content of the e-learning course, it's because of the teacher's influence. | MSLQ_30 | control beliefs |  |  |  |  |  |
| When reading or submitting assignments on the e-learning platform, I feel that my heart beats fast. | MSLQ_31 | emotionalism |  |  |  |  |  |
| I am confident that I can master the course content taught on the e-learning platform. | MSLQ_32 | self-efficacy |  |  |  |  |  |
| If I learned the content of the e-learning course, it's because the course was easy to learn. | MSLQ_33 | control beliefs |  |  |  |  |  |
| I want to perform well in the e-learning course in my class because it's important for me to demonstrate my abilities to family and others. | MSLQ_34 | extrinsic goal orientation |  |  |  |  |  |
| Considering the difficulty of the e-learning course, the guidance of the teacher, and my personal learning abilities, I think I will perform well. | MSLQ_35 | expectancy |  |  |  |  |  |
| **C.    Approaches to Learning and Studying Inventory, ALSI** |  |  |  |  |  |  |  |
| I often struggle to understand the content and logic of e-learning platform courses. | ALSI_01 | Surface approach |  |  |  |  |  |
| After completing the e-learning assignments, I will review my reasoning process and ensure its logical coherence. | ALSI_02 | Monitoring studying |  |  |  |  |  |
| I'm beginning to understand the significance of learning through the e-learning platform. | ALSI_03 | Deep approach |  |  |  |  |  |
| I put a lot of effort into my studies on the e-learning platform. | ALSI_04 | Effort management |  |  |  |  |  |
| What I've learned on the e-learning platform often lingers in my mind. | ALSI_05 | Surface approach |  |  |  |  |  |
| The knowledge I acquire on the e-learning platform can be connected in practical applications | ALSI_06 | Deep approach |  |  |  |  |  |
| My learning approach on the e-learning platform is systematic and organized. | ALSI_07 | Organized studying |  |  |  |  |  |
| I often have many thoughts in e-learning platform courses that lead me into long periods of contemplation. | ALSI_08 | Deep approach |  |  |  |  |  |
| After class, I still use the e-learning platform materials for review. | ALSI_09 | Deep approach |  |  |  |  |  |
| When I'm reviewing information on the e-learning platform, I can quickly synthesize my own perspectives. | ALSI_10 | Monitoring studying |  |  |  |  |  |
| I will plan my e-learning platform study time and make the most of it. | ALSI_11 | Organized studying |  |  |  |  |  |
| For me, both the theoretical aspects of static materials and a deep understanding of dynamic materials on the e-learning platform are important. | ALSI_12 | Deep approach |  |  |  |  |  |
| I will not doubt what I have learned on the e-learning platform. | ALSI_13 | Surface approach |  |  |  |  |  |
| I will search for relevant information outside of the e-learning platform. | ALSI_14 | Monitoring studying |  |  |  |  |  |
| Focusing on e-learning is not a problem for me unless I am really tired. | ALSI_15 | Effort management |  |  |  |  |  |
| When reading a unit on the e-learning platform, I try to understand what the author wants to convey. | ALSI_16 | Deep approach |  |  |  |  |  |
| For the course content on the e-learning platform, if I haven't reached my set goals, I will keep going on. | ALSI_17 | Surface approach |  |  |  |  |  |
| If there is knowledge on the e-learning platform that I can't understand, I will try different methods to learn. | ALSI_18 | Monitoring studying |  |  |  |  |  |
| **D.    Maslach burnout inventory-student survey, MBI-SS** |  |  |  |  |  |  |  |
| E-learning courses can make me feel emotionally down about learning. | MBI_01 | Emotional exhaustion |  |  |  |  |  |
| After completing a full day of e-learning courses, I feel completely exhausted. | MBI_02 | Cynicism |  |  |  |  |  |
| When I wake up in the morning and think about a full day of e-learning courses, I feel tired, and I want to skip class. | MBI_03 | Academic efficacy |  |  |  |  |  |
| I feel tired of e-learning platform courses. | MBI_04 | Emotional exhaustion |  |  |  |  |  |
| E-learning platform learning is really tiring for me. | MBI_05 | Cynicism |  |  |  |  |  |
| My enthusiasm for the Medical Technology program disappeared when I started using the e-learning platform for courses. | MBI_06 | Academic efficacy |  |  |  |  |  |
| I began to feel negative about taking e-learning courses. | MBI_07 | Emotional exhaustion |  |  |  |  |  |
| E-learning has become a source of stress for me. | MBI_08 | Academic efficacy |  |  |  |  |  |
| I doubt the significance of learning e-learning courses. | MBI_09 | Academic efficacy |  |  |  |  |  |
| I can effectively address academic problems using the e-learning platform courses. | MBI_10 | Emotional exhaustion |  |  |  |  |  |
| I believe that participating in e-learning courses is helpful for me. | MBI_11 | Cynicism |  |  |  |  |  |
| In my opinion, I am a good student. | MBI_12 | Academic efficacy |  |  |  |  |  |
| I feel excited when I achieve my learning goals in E-learning courses. | MBI_13 | Emotional exhaustion |  |  |  |  |  |
| I feel like I've learned many interesting things in E-learning courses. | MBI_14 | Cynicism |  |  |  |  |  |
| I have confidence that I can effectively absorb the content of e-learning courses. | MBI_15 | Academic efficacy |  |  |  |  |  |
